# The Anaphylactoid Syndrome of Pregnancy: Two Autopsy Cases

**DOI:** 10.7759/cureus.45145

**Published:** 2023-09-13

**Authors:** Ioannis Plantzas, Athina Tousia, Dimitrios Vlachodimitropoulos, Maria Piagkou, Nikolaos Goutas, George Tsakotos, George Triantafyllou, Evangelos Plantzas, Emmanouil Sakelliadis

**Affiliations:** 1 Forensic Medicine and Toxicology, National and Kapodistrian University of Athens, Athens, GRC; 2 Anatomy, National and Kapodistrian University of Athens, Athens, GRC

**Keywords:** autopsy, fetus, death, diffuse intravascular coagulation, lungs, anaphylactoid syndrome of pregnancy, amniotic fluid embolism

## Abstract

The anaphylactoid syndrome of pregnancy (ASP) is a rare emergency with significant mortality and morbidity, in which the amniotic fluid and fetal cells enter the maternal circulation leading to respiratory failure, altered mental status, hypotension, and disseminated intravascular coagulation. The term ASP was recently introduced to replace the term amniotic fluid embolism since the clinical manifestations of the entity were more similar to a septic or anaphylactic shock rather than that of an embolic event. Two autopsy cases are described, regarding a 35-year-old gravida 2 para 1 and a 34-year-old gravida 1 para 0, where the cause of death was determined to be ASP.

## Introduction

The anaphylactoid syndrome of pregnancy (ASP) is a rare obstetric emergency occurring due to the entrance of amniotic fluid and fetal matter into the maternal circulation, leading to sequelae [1]. The term ASP was introduced to replace the term amniotic fluid embolism (AFE) since the clinical manifestations of the entity were more similar to a septic or anaphylactic shock rather than that of an embolic event [2]. ASP’s four clinical signs are respiratory failure, altered mental status, hypotension, and disseminated intravascular coagulation (DIC). The aforementioned are a result of cytokine release due to the entrance of amniotic fluid into the mother’s circulation, which through complex immune pathways, will lead to clotting cascade dysfunctions [1]. The ASP diagnosis has traditionally been made at autopsy when fetal squamous cells are identified in the maternal pulmonary circulation [3], although clinically, it is considered a diagnosis of exclusion [1]. The fundamental pathophysiology difference between the two is that ASP consists of a blockage event in the pulmonary system due to an embolus while ASP describes a proinflammatory response after the release of cytokines and arachidonic acid metabolites into the bloodstream, resulting in the entity’s clinical manifestations [1]. The incidence of ASP is estimated at one case per 8000 to 30,000 pregnancies. The prognosis after ASP has been relatively poor, as maternal mortality ranges between 13.5% and 44% while perinatal mortality ranges between 7% and 38% [4]. Some of the proposed risk factors for this emergency are the mother’s older age, multiparity, intense contractions during labor, abdominal trauma, cesarean section, induction of labor, placenta previa, eclampsia, uterus or cervix tears, and the placenta’s early separation from the uterus wall. In addition, the fetus's distress, death, and male gender are risk factors for the development of ASP [5]. ASP should be considered in the differential diagnosis, along with myocardial infarction, anaphylaxis, aortic dissection, sepsis, pulmonary air embolism, aspiration, eclampsia, toxic reactions to anesthetic drugs, and hemorrhagic shock [6].

In the current autopsy reports, two fatal cases (one including both the mother’s and fetus’ death and the other only the mother’s) of ASP with DIC involvement are described, focusing on histopathology findings.

## Case presentation

Case 1

A 35-year-old, G2 P1 on the 36th gestational week visited the hospital for a scheduled prenatal appointment. She has noticed an absence of fetal movements for the last 48 hours. The woman had a history of one previous, expected, spontaneous vaginal delivery, 15 years ago (weight 2750 gr). No significant health conditions or family history were present. Intrauterine fetal death was confirmed via ultrasound. Labor induction is followed to remove the fetus. During the active labor phase of cervical dilation (4-5 cm) and weak uterine contractions, spontaneous rupture of membranes took place, resulting in the outflow of a colored and malodorous amniotic fluid. During the dead fetus expulsion (weight 2030 gr) through vacuum aspiration, the mother presented loss of consciousness. The intrauterine death was provoked by six windings of the umbilical cord around the uterus's neck. Resuscitation was started immediately. Blood investigation during resuscitation didn’t reveal anemia (Hematocrit: 40.3%, Hemoglobin: 12,3 g/dL). Minutes later, she developed vaginal bleeding due to uterine atony. Medication administration, uterine tamponade, uterine massage, and blood transfusion were performed and 35 minutes later, hemorrhage control was achieved. Bleeding reappeared half an hour later, which was initially, but unsuccessfully, treated conservatively. The woman was then admitted to the operating room, where uterine vessel ligation and hysterectomy were performed. During wound closure, the woman suffered a cardiac arrest. After resuscitation attempts, the woman succumbed approximately an hour later.

**Figure 1:**
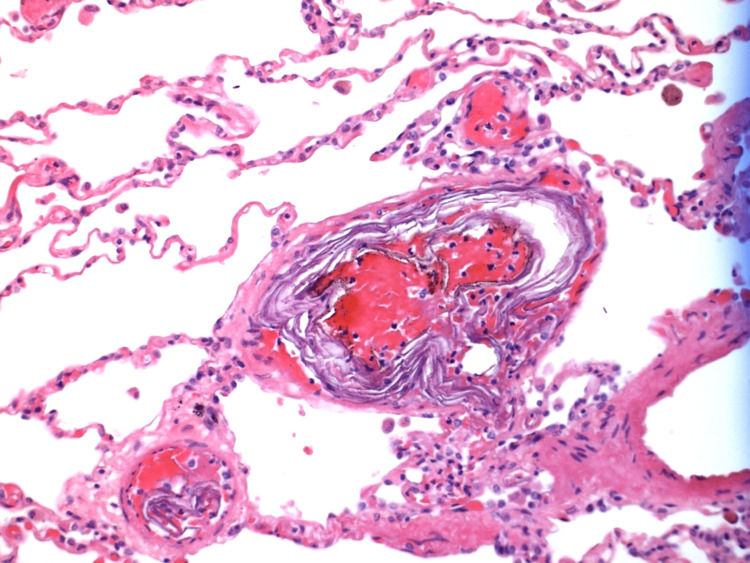
Findings in the lumen of a small vessel from Case 1 Fibrin thrombi inside a small vessel (magnification x400)

**Figure 2:**
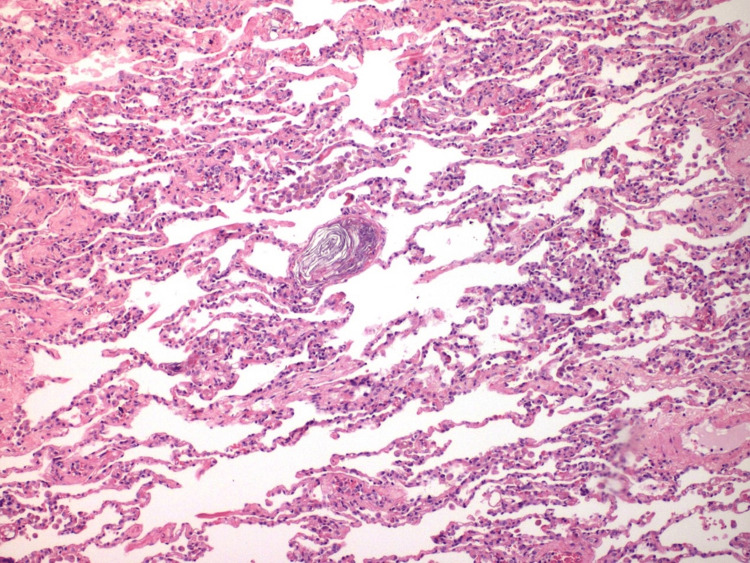
Findings in the lumen of small vessels of the lung tissue from Case 1 Fibrin thrombi inside the lumen of the small vessels of the lung (magnification x100)

According to the fetus’ autopsy maceration was witnessed, a finding indicative of intrauterine death at least two days before. Signs suggestive of intrauterine stress, with meconium exit, intense hyperemia, and cerebral anoxia, due to the umbilical cord incident were also witnessed. Finally, there were no congenital abnormalities - dysplasia or pathologies of the fetus. According to the mother’s autopsy, multiple organs’ histopathology findings presented hemorrhagic and inflammatory infiltrate, edema, amorphous eosinophilic material, as well as fibrin deposition in small and medium-sized vessels, suggestive of DIC (Figure 1). The appearance of mucus, fetal epithelial squames, and meconium in the lung vessels suggested the ASP diagnosis (Figure 2). Hemosiderin granules were also present. The cervix presented multiple impressions, which confirmed the multiple umbilical cord windings.

Case 2

A 34-year-old G1P0 woman, in the 40th gestational week, was admitted to the hospital. No significant health conditions or family history were present. After epidural anesthesia, labor induction took place. The newborn weighed 4020 gr. Ten minutes after delivery, she developed excessive bleeding due to uterine atony. Conservative treatment consisting of uterus tamponade was performed, followed by uterus vessel ligation. After suffering a cardiac arrest, the woman succumbed.

**Figure 3:**
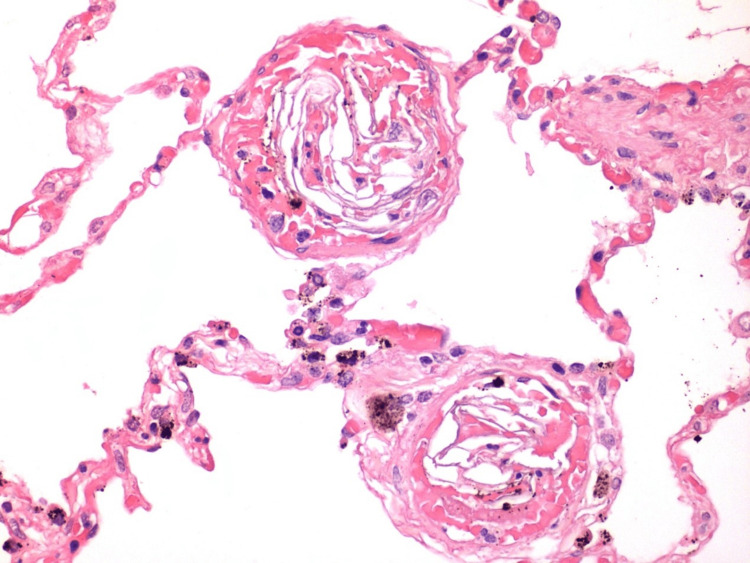
Findings in the lumen of the lung vessels from Case 2 Keratin scales in the lumen of lung vessels (magnification x400)

According to the woman’s autopsy, the histopathological examination of the lungs revealed multiple thrombi in the small vessels and polymorphonuclear cells’ mobilization. The alveoli included macrophages that phagocyte hemosiderin. Keratin flakes were included in the lumen of the small vessels due to the ASP (Figure 3). In the kidneys, fibrin thrombi were identified in the vessels of the renal hilum because of DIC.

## Discussion

Specific criteria must be presented to clinically diagnose ASP (Table 1) [7].

**Table 1:** The international criteria for the diagnosis of anaphylactoid syndrome of pregnancy (ASP)/amniotic fluid embolism (AFE) Source: [7]

International Criteria for Diagnosis ASP/AFE
Sudden onset of cardiopulmonary arrest or both hypotension and respiratory compromise
Documentation of overt disseminated intravascular coagulation after the appearance of initial signs and symptoms
Clinical onset during labor or within 30 minutes of the passing of the placenta
Lack of fever during labor

Both cases did not have a history of fever and presented cardiopulmonary arrest minutes after delivery. Based on these criteria, due to the lack of provided information about investigation reports suggestive of DIC during hospitalization, criteria number 2 cannot be further discussed. In the first case, according to the literature, the unique included risk factor was the fetus’ death [5]. In terms of the clinical signs of AFE, uterus atony, cardiac arrest, and severe hemorrhage (DIC) were present in both cases (Table 2) [8].

**Table 2:** Signs and symptoms indicative of possible anaphylactoid syndrome of pregnancy (ASP)/amniotic fluid embolism (AFE) Source: [8]

Acute dyspnea or sudden, agitation, sudden chills, shivering, sweating, coughing, and anxiety are common premonitory symptoms. Labored breathing and tachypnea may occur
Cough
Altered mental status
Rapid decline in pulse oximetry values or sudden absence or decrease in end-tidal carbon dioxide may be apparent
Hypotension
Cyanosis
Fetal bradycardia
Encephalopathy associated with AFE
Uterine atony
Acute pulmonary hypertension and vasospasm results in right ventricular failure, hypoxia, and cardiac arrest
Coagulopathy or severe hemorrhage

In the vast majority of ASP cases (83%), DIC is identified, with the latter occurring as quickly as 10-30 min from the onset of ASP symptoms [5]. Coagulopathy is believed to occur after the procoagulant components of the amniotic fluid that enter the bloodstream cause a clotting cascade dysfunction leading to severe fibrinolytic activity and finally DIC [1]. The histopathology findings of both cases suggested DIC development (in the 2nd case, thrombi were also observed at the renal hilum vessels). The classic DIC histopathological finding is the appearance of microvascular fibrin deposition [9]. Both cases’ findings included the appearance of degenerate fetal epithelial cells, as from keratin scales. According to the literature, findings of amniotic fluid and fetal cells in the lungs establish the ASP diagnosis histopathologically [2]. Another histopathological finding, in both cases, is the appearance of macrophages that phagocyte hemosiderin, which, in the literature, are related to the presence of heart failure [10]. Heart failure occurs since the entity itself leads to left-sided heart failure through pulmonary edema because of both the emboli event and ASP’s complex inflammatory response pathways [1]. Both cases experienced uterine atony, a condition that is associated with DIC [11]. Uterine atony is the most common etiology of postpartum hemorrhage [12]. Risk factors for its occurrence include induced or augmented labor, polyhydramnios, multiple gestations, chorioamnionitis, cesarean delivery, Hispanic ethnicity, prolonged labor or prolonged second stage of labor, preeclampsia, macrosomia, magnesium therapy, and extremes of maternal age [13]. In the first case, according to the provided blood test results, there were no signs of anemia during resuscitation, ruling out the hemorrhagic shock as the cause of cardiopulmonary arrest. In the atypical form of ASP, like the second case, cardiopulmonary collapse does not occur, and the initial symptom is a life-threatening hemorrhage due to the DIC appearance [14]. Since squamous cells, trophoblasts, and other fetal debris can be found in the central circulation of women with conditions other than ASP uterine atony associated with DIC should always be included in the differential diagnosis of similar cases [15]. Precision in reporting the timeline of clinical information is important to shape an accurate differential diagnosis.

## Conclusions

ASP continues to be a worrying entity in obstetric medicine due to its clinical unpredictability and associated maternal and neonatal mortality. Early diagnosis and supportive treatment may improve patient outcomes, although the entity’s mortality rates are still considerably high. It is essential for physicians to report such cases with precision regarding the diagnostic investigation results and timeline, not only to assist the utilization of diagnostic and therapeutic tools but also to support a more accurate estimation of the prevalence and mortality of ASP. Concerning the postmortem investigation, a forensic pathologist should be aware of any possible therapeutic intervention that may be performed, as knowledge of these parameters should help reduce any confusion during the postmortem examination and thus aid in establishing the correct cause of death. As always, histopathologic examination of tissues collected during the postmortem examination proves to be an invaluable tool to confirm or deny macroscopical diagnosis and thus should be performed in such cases. Finally, it is also important for pathologists to publish ASP cases so that evidence about the disorder can be established, resulting in a better understanding of the condition itself.
